# Sex with sweethearts: Exploring factors associated with inconsistent condom use among unmarried female entertainment workers in Cambodia

**DOI:** 10.1186/s12879-016-2101-2

**Published:** 2017-01-05

**Authors:** Siyan Yi, Sovannary Tuot, Pheak Chhoun, Khuondyla Pal, Chanrith Ngin, Kolab Chhim, Carinne Brody

**Affiliations:** 1 KHANA Center for Population Health Research, No. 33, Street 71, Tonle Bassac, Chamkar Mon, Phnom Penh, Cambodia; 2 Center for Global Health Research, Public Health Program, Touro University California, Vallejo, USA; 3 HIV/AIDS Flagship Project, KHANA, Phnom Penh, Cambodia

**Keywords:** Condom use, Female entertainment workers (FEWs), Sexual and reproductive health, Sweetheart, Cambodia

## Abstract

**Background:**

Despite the success in promoting condom use in commercial relationships, condom use with regular, noncommercial partners remains low among key populations in Cambodia. This study explores factors associated inconsistent condom use with sweethearts among unmarried sexually active female entertainment workers (FEWs).

**Methods:**

In 2014, the probability proportional to size sampling method was used to randomly select 204 FEWs from entertainment venues in Phnom Penh and Siem Reap for face-to-face interviews. Multivariate logistic regression analysis was conducted to examine independent determinants of inconsistent condom use.

**Results:**

Of total, 31.4% of the respondents reported consistent condom use with sweethearts in the past three months. After adjustment, respondents who reported inconsistent condom use with sweethearts remained significantly less likely to report having received any form of sexual and reproductive health education (AOR = 0.49, 95% CI = 0.22–0.99), but more likely to report having been tested for HIV in the past six months (AOR = 2.19, 95% CI = 1.03–4.65). They were significantly more likely to report having used higher amount of alcohol in the past three months (AOR = 1.29, 95% CI = 1.01–1.99) and currently using a contraceptive method other than condoms such as pills (AOR = 4.46, 95% CI = 1.34–10.52) or other methods (AOR = 9.75, 95% CI = 2.07–9.86).

**Conclusions:**

The rate of consistent condom use in romantic relationships among unmarried FEWs in this study is considerably low. The importance of consistent condom use with regular, non-commercial partners should be emphasized in the education sessions and materials, particularly for FEWs who use non-barrier contraceptive methods.

## Background

In the Cambodian context, female entertainment workers (FEWs) refer to women working in entertainment venues such as massage parlors, night clubs, karaoke bars, beer gardens, etc. and who may or may not be involved in transactional sex [1]. The context of transactional sex in Cambodia has been dramatically altered by the introduction of the “Law on Suppression of Human Trafficking and Sexual Exploitation” in 2008 [2]. Many brothels closed down, and transactional sex has shifted to entertainment venues or other informal and hidden settings such as streets and parks. The lines between direct and indirect sex work have become less clear, and an increase in indirect transactional relationships, such as sweethearts, has been documented [3].

“Sweethearts,” as they are called locally, involve romantic relationships and include normative lack of condom use as displays of trust and intimacy as well as indirect transactional sex through dinner dates, gifts or shopping trips [3]. For FEWs, a sweetheart is typically a boyfriend and/or regular client [4]. They are “a partner from a non-commercial, non-marital sexual relationship that possesses a certain degree of affection and trust” [5]. A sweetheart could give regular gifts and other forms of support. Anecdotal evidence indicates that many sweethearts of FEWs are previous clients [6, 7]. Their relationship has become intimate over time, which enables a client to become a regular client and then a sweetheart. Some sweethearts become FEWs’ cohabitating partner or spouse [6, 7].

Marginalized communities had been affected by the global financial crisis in 2008, which led to the closure of several garment factories, with anecdotal evidence indicating that a number of female garment workers became workers in the entertainment industry. The National Center for HIV/AIDS, Dermatology and STD (NCHADS) estimated that there were 35,000 FEWs in Cambodia in 2012, of whom 60% were living in Phnom Penh [5]. In a 2012 study, all FEWs reported multiple, concurrent partners, including clients and sweethearts [4]. The number of clients for FEWs ranged from four to five a day—particularly for FEWs who worked in massage parlors and brothels—to one a month [4]. A 2010 assessment reported that FEWs were most likely to have recent sex with a client (38%), compared to a sweetheart (31%) or spouse (24%) [8]. On living arrangements, 7% of FEWs lived with a sweetheart [8].

The number of FEWs who have sweethearts is high, and most of them have active sex with their sweethearts. A 2015 study revealed that 60% of FEWs reported having one or more sweethearts in the past year, and about 85% of those with sweethearts reported having sex with them in the past three months [5]. In the 2010 assessment, about 20% of FEWs had sweethearts only in the past three months [8].

While the dramatic reduction of HIV prevalence in the general population was a cause for celebration in Cambodia, a great deal of challenges remain in reducing the prevalence of HIV and sexually transmitted infections (STIs) as well as in addressing other sexual and reproductive health (SRH) issues among FEWs who engage in transactional sex [9].

FEWs are at increased risk of both HIV/STI infections and poor SRH outcomes because of their high likelihood of involvement in direct or indirect transactional sex [1]. A recent study reported that HIV prevalence among this group is alarmingly high at 9.8% [10], compared to 0.6% in the general population [9]. According to the midterm data of the Sustainable Action against HIV and AIDS in Communities (SAHACOM) project, which provides comprehensive HIV and SRH services to FEWs in Cambodia, approximately 40% of FEWs reported having at least one STI symptom in the past three months [11]. Moreover, we recently found that 54% of FEWs reported at least one induced abortion during their lifetime, and 33% while working as a FEW [12]. The rates of consistent condom use with commercial and non-commercial partners in the past three months were 79 and 31%, respectively [13]. Through the five years of the SAHACOM lifespan, the rates of condom use with both commercial and non-commercial partners were not appreciably improved [12]. Inconsistent condom use with non-commercial partners among FEWs signifies a need for refinement in interventions, given their ubiquitous practice of having such partners and their high HIV prevalence.

Exploring factors associated with inconsistent condom use with non-commercial partners among FEWs is important for prevention programs to eliminate new HIV infections and improve SRH outcomes such as STIs, unwanted pregnancies and subsequent induced abortions. Several studies have been conducted to explore factors associated with condom use among female sex workers (FSWs) in different settings. Guided by the Health Belief Model, Zhao and colleagues found that in China condom use was associated with self-efficacy and perceived benefits, and lack of use was associated with perceived barriers to condom use [14]. Excessive alcohol drinking has been found to be associated with both unprotected sex and a history of STIs among FSWs in China [15] and Uganda [16]. In Bolivia, women who used non-barrier modern contraception were less likely to consistently use condoms with non-commercial partners than non-users [17]. In terms of sexual behaviors, the risk of inconsistent condom use decreased when the number of sexual partners increased [18].

FEWs in the wake of the brothel ban in Cambodia are a mixed population of women who might be involved in direct or indirect sex work, while also have romantic relationships. The lifestyle of FEWs thus is unique to the Cambodian context, and their sexual activities are more complex than the lifestyles and behaviors of FSWs in other countries. While evidence that reports factors associated with condom use among FSWs is important, there is a need to understand determinants of condom use among FEWs in Cambodia. Most studies of condom use among FSWs examined commercial relationships [14, 16, 18], while the rates of consistent condom use with non-commercial partners are low and have remained low in the past several years in almost all settings, including Cambodia [12, 13, 19, 20]. This study was therefore conducted to explore factors associated with inconsistent condom use with sweethearts among FEWs in Cambodia. In our study, we defined commercial partners or clients as those who paid (either money or a gift) for sex with FEWs but did not engage in a romantic relationship, while we connoted non-commercial partners or sweethearts of FEWs by following the preceding elaboration.

## Methods

This study was conducted as part of the impact evaluation of the SAHACOM project. Data were derived from the end-line survey conducted in April and May 2014. Details of this survey have been published elsewhere [12, 13].

### Participants and sampling

Face-to-face interviews were conducted with 667 FEWs randomly selected from entertainment venues under the program coverage of the SAHACOM project in Phnom Penh and Siem Reap. The population of FEWs in these two provinces represented approximately 70% of the total population of FEWs in Cambodia. The probability proportional to size sampling method was used to decide the number of FEWs in each province, and venues were then randomly selected. A proportionate number of participants were randomly selected from a name list of FEWs of each selected venue. A FEW would be included in the study if she was: (1) biologically female, (2) at least 18 years of age, (3) able to present herself on the day of the interview and (4) able to provide consent to participate in the study.

### Data collection training and procedure

All interviewers and field supervisors were trained for three days. The training covered a review of the study protocol, informed consent process, interview techniques and confidentiality. The research teams were also equipped with quality control and problem solving skills. Regular review sessions were encouraged to be performed among research team leaders and interviewers to follow up the progress and communicate any issues occurring during the data collection.

### Questionnaire development

A structured questionnaire was initially developed in English, translated into Khmer and back-translated into English. A pilot study was conducted among a random sample of 20 FEWs, and the questionnaire was modified accordingly.

The questionnaire was developed using existing tools from previous studies with same population [11], the 2010 Demographic and Health Survey in Cambodia [21], as well as from other relevant studies in Cambodia [22–24]. Socio-demographic characteristics included marital status, age, formal education, average monthly income, living arrangements, types of venues at which they were working and duration of time they had worked in the entertainment industry and at their current establishment.

Several questions were employed to measure sexual behaviors and condom use in different types of sexual relationships in the past three months. The variables included the number of sex partners (both clients and sweethearts) and condom use with both types of sex partners. Condom use was measured using a scale with six-point response options ranging from (1) “always” to (6) “never.” Those respondents who answered “always” to the questions were labeled as consistent condom users. Respondents were also asked if they were able to find condoms when needed in the past three months (0 = no, 1 = yes).

Regarding substance use, participants were questioned about the use of alcohol (at least a full glass of beer, wine or liquor) and illicit drugs (including methamphetamine, heroin, ecstasy, inhalants, cocaine or marijuana) in the past three months. Response options were yes or no. Those who reported drinking alcohol in the past three months were also questioned about the average amount they drank per day (number of cans for beer and glasses for wine). We also collected information on the history of contraceptive use, pregnancy, induced abortion and STIs as well as HIV and SRH education they had received in the past six months.

To measure mental health, we adapted a short version of the General Health Questionnaire (GHQ-12) [25] with four response options of “0 = less than usual,” “1 = no more than usual,” “2 = rather more than usual” or “3 = much more than usual.” The scoring method ‘0-0-1-1’ was used because it is believed to reduce any biases caused by respondents who tend to choose responses 0 and 3 or 1 and 2 [26]. To measure the level of mental disorder, the mean score for the study population [4.1 (SD = 2.7)] was used as the cut-off; scores above 4 were considered “high”, and ﻿4 or below were considered low [27]. The Cronbach’s α of GHQ-12 scale among FEWs in this study was 0.70.

A 12-item scale was adapted from a previous study to assess HIV knowledge [28]. The response options were ‘0 = No,’ ‘1 = yes’ or ‘2 = don’t know.’ The total score of the scale was the sum of correct responses, with ‘don’t know’ responses scored as incorrect. The Cronbach’s α of this scale among FEWs in this study was 0.72.

### Data analyses

EpiData version 3 was used for double data entry (Odense, Denmark). Data analyses were performed taking into account the sampling weight of sampling-size differences of FEWs population although the sampling design was self-weighted within each site [29]. In bivariate analyses, Student’s *t*-test was used for continuous variables, and Chi-square test or Fisher’s exact test was used as appropriate for categorical variables to compare socio-demographic characteristics, sexual behaviors, history of SRH, substance use, HIV knowledge and mental health (GHQ12) among respondents who reported consistent condom use and inconsistent condom use with one or more sweethearts in the past three months. A multivariate logistic regression model was developed. We simultaneously included all variables associated with condom use in bivariate analyses at a level of *p* < 0.2 in the model. Adjusted odds ratio (AOR), 95% confidence intervals (CI) and *p*-values were calculated. Two-sided *p*-value <0.05 was used to determine statistical significance. SPSS version 22 (IBM Corporation, New York, USA) was used for all analyses.

## Results

### Characteristics of respondents

This study included 204 FEWs (30.6% of the total sample) who reported having sexual intercourse with one or more sweethearts in the past three months, with a mean age of 25.7 years (SD = 5.4 years). Only 31.4% reported consistent condom use with their sweethearts in the past three months. As shown in Table 1, the majority (77.9%) of the respondents were recruited from Phnom Penh, and more than two-thirds worked in Karaoke parlors (53.9%) and restaurants (25.5%). The rate of consistent condom use was significantly higher among respondents who reported working in the entertainment industry for less than 28 months (37.1 vs. 21.4%, *p* = 0.02), and in the current establishment for less than 18 months (36.0 vs. 21.9%, *p* = 0.04) compared to their comparison group. No significant differences were found in the comparisons of other socio-demographic characteristics, self-perception of HIV risk, mental health and levels of HIV knowledge.

**Table 1:** Comparisons of characteristics of female entertainment workers who reported consistent and inconsistent condom use with sweethearts

Socio-economic characteristics	Total (*n* = 204)	Condom use in the past 3 months		
		Consistent (*n* = 64)	Inconsistent (*n* = 140)	*p*-value^*^
Province				0.97
Phnom Penh	159 (77.9)	50 (31.4)	109 (68.6)	
Siem Reap	45 (22.1)	14 (31.1)	31 (68.9)	
Mean age (in year)	25.7 ± 5.4	25.3 ± 5.2	25.9 ± 5.4	0.47
Years of formal education completed	6.4 ± 2.9	6.0 ± 2.6	6.6 ± 3.0	0.17
Currently place of employment				0.79
Karaoke parlor	110 (53.9)	33 (30.0)	77 (70.0)	
Restaurant	52 (25.5)	16 (30.8)	36 (69.2)	
Other	42 (20.6)	15 (35.7)	27 (64.3)	
Working duration in this career^†^				0.02
< 28 months	132 (65.3)	49 (37.1)	83 (62.9)	
≥ 28 months	70 (34.7)	15 (21.4)	55 (78.6)	
Working duration for the current establishment^†^				0.04
< 18 months	139 (68.5)	50 (36.0)	89 (64.0)	
≥ 18 months	64 (31.5)	14 (21.9)	50 (78.1)	
Mean monthly income (in US$)	232 ± 155	238 ± 168	229 ± 149	0.71
Received HIV education (in past 6 months)				0.35
No	60 (29.4)	16 (26.7)	44 (73.3)	
Yes	144 (70.6)	48 (33.3)	96 (66.7)	
Tested for HIV in the past 6 months				0.10
No	85 (41.7)	32 (37.6)	53 (62.4)	
Yes	119 (58.3)	32 (26.9)	87 (73.1)	
Self-perception of HIV risk compared to the general population				0.26
Higher	54 (26.5)	13 (24.1)	41 (75.9)	
Same	40 (19.6)	11 (27.5)	29 (72.5)	
Lower	83 (40.7)	28 (33.7)	55 (66.3)	
Don’t know	27 (13.2)	12 (44.4)	15 (55.6)	
Total score of General Health Questionnaire (GHQ12)^†^				0.24
≤ 3 (lower than the average level)	95 (47.0)	33 (34.7)	62 (65.3)	
≥ 4 (higher than the average level)	107 (53.0)	29 (27.1)	78 (72.9)	
Mean score of HIV knowledge	21.2 ± 1.9	21.1 ± 2.2	21.5 ± 1.8	0.17

Values are number (%) for categorical variables and mean ± SD for continuous variables

^*^Chi-square test or Fisher’s exact test was used as appropriate for categorical variables and Student’s *t*-test was used for continuous variables

^†^Mean values were used to categorize the participants

### Substance use, sexual behaviors and SRH

Comparisons of substance use, sexual behaviors and SRH among respondents who reported consistent and inconsistent condom use with one or more sweethearts in the past three months are shown in Table 2. The average amount of alcohol consumed per day was significantly higher among respondents who reported inconsistent condom use (mean = 10.3 cans/glasses, SD = 12.2) than among those who reported consistent condom use (mean = 7.0 cans/glasses, SD = 7.9) with sweethearts (*p* = 0.03). Respondents who reported consistent condom use with sweethearts were significantly more likely to report consistent condom use with clients in the past three months (88.5 vs. 64.4%, *p* = 0.02). Regarding contraceptive use, respondents who reported consistent condom use with sweethearts were significantly more likely to report current use of a modern contraceptive method (71.9 vs. 48.2%, *p* = 0.002), and using condoms as the main contraceptive method (87.2 vs. 32.8%, *p* < 0.001). Moreover, respondents who reported consistent condom use with sweethearts were significantly more likely to report having received some form of SRH education in the past six months (70.3% vs. 57.6%, *p* = 0.04).

**Table 2:** Comparisons of substance use, sexual behaviors and SRH among FEWs who reported consistent and inconsistent condom use with sweethearts

	Total (*n* = 204)	Condom use in the past 3 months		
		Consistent (*n* = 64)	Inconsistent (*n* = 140)	*p*-value^*^
Substance use in the past 3 months				
Drunk at least a full glass of alcohol	193 (94.6)	59 (92.2)	134 (95.7)	0.30
Mean number of days getting drunk in past month	23.0 ± 11.0	23.4 ± 10.6	22.8 ± 10.6	0.75
Mean amount of alcohol per day (cans, glasses)	8.0 ± 9.5	7.0 ± 7.9	10.3 ± 12.2	0.03
Self-perception of level of alcohol drinking				0.40
Non-drinkers	16 (8.3)	5 (8.5)	11 (8.2)	
Social drinkers^†^	159 (82.4)	46 (78.0)	113 (84.3)	
Heavy drinkers^‡^	18 (9.3)	8 (13.6)	10 (7.5)	
Used any kinds of illicit drugs	9 (4.4)	1 (1.6)	8 (5.7)	0.18
Sexual behaviors in the past 3 months				
Mean number of sex partners	4.6 ± 8.4	5.4 ± 9.7	4.1 ± 7.8	0.31
Had sex with clients in exchange for money/gifts	68 (34.2)	23 (37.7)	45 (32.6)	0.49
Mean number of clients	3.9 ± 4.5	4.0 ± 6.2	3.7 ± 3.4	0.79
Able to find condom when needed	162 (79.4)	53 (82.8)	109 (77.9)	0.42
Always used condom with clients	52 (72.2)	20 (88.5)	29 (64.4)	0.02
Sexual reproductive health				
Currently using a contraceptive method	113 (55.7)	46 (71.9)	67 (48.2)	0.002
Type of contraceptive method being used				<0.001
Pills	22 (19.8)	4 (8.5)	18 (28.1)	
Condom	62 (55.9)	41 (87.2)	21 (32.8)	
Other (injection, IUD, implant, natural)	27 (24.3)	2 (4.3)	25 (39.1)	
Received SRH education in the past 6 months	125 (61.6)	45 (70.3)	80 (57.6)	0.04
Diagnosed with an STI in the past 6 months	52 (25.5)	14 (21.9)	38 (27.1)	0.42
Had an abortion in the past 12 months	53 (26.0)	12 (18.8)	41 (29.3)	0.11

Values are number (%) for categorical variables and mean ± SD for continuous variables

Abbreviations: *FEWs* female entertainment workers, *IUD* intrauterine devices *SRH* sexual and reproductive health, *STI* sexually transmitted infection

^*^Chi-square test or Fisher’s exact test was used as appropriate for categorical variables and Student’s *t*-test was used for continuous variables

^†^‘Social drinkers’ were defined as people who drink alcohol chiefly on social occasions and only in moderate quantities

^‡^‘Heavy drinkers’ were defined as men who drink more than 15 drinks or women who drink more than eight drinks in a week

### Results of multivariate analyses

Table 3 shows factors that remained significantly associated with inconsistent condom use with sweethearts in the past three months after controlling for other covariates in a multivariate logistic regression model. Respondents who reported inconsistent condom use with sweethearts remained significantly less likely to report having received some form of SRH education (AOR = 0.49, 95% CI = 0.22–0.99), but more likely to report having been tested for HIV (AOR = 2.19, 95% CI = 1.03–4.65) in the past six months. They were significantly more likely to report having used higher amount of alcohol (AOR = 1.29, 95% CI = 1.01–1.99) and currently using contraceptive methods other than condoms such as pills (AOR = 4.46, 95% CI = 1.34–10.52) or other methods (AOR = 9.75, 95% CI = 2.07–9.86).

**Table 3:** Factors associated with inconsistent condom use with sweethearts among FEWs in multiple logistic regression model

Variables in the final model^*^	Inconsistent condom use in the past 3 months	
	AOR (95% CI)	*p*-value
Received sexual and reproductive health education in the past 6 months		0.04
No	Reference	
Yes	0.49 (0.22–0.99)	
Tested for HIV in the past 6 months		0.03
No	Reference	
Yes	2.19 (1.03–4.65)	
Higher amount of alcohol use per day (past 3 months)	1.29 (1.01–1.99)	0.04
Contraceptive method being currently used		
Condom	Reference	
Pills	4.46 (1.34–10.52)	0.001
Other^†^	9.75 (2.07–9.86)	<0.001

Abbreviations: *AOR* adjusted odds ratio, *CI* confidence interval, *FEW* female entertainment worker

^*^Other variables in the model included education, working duration in the entertainment career and establishment, currently using a contraceptive method, condom use with clients and history of abortion

^†^Other included injection, intrauterine devices, implant, calendar and natural methods

## Discussion

In Cambodia, success in increasing condom use with commercial partners in key populations and significant reduction of HIV prevalence in the general population are attributed to the national level programmatic efforts made in the past decades. However, this study found that the rate of consistent condom use with sweethearts remains unacceptably low among FEWs (31.4% of those having sex with sweethearts in the past three months). This finding is in line with findings in several studies in other countries, which found that the rates of consistent condom use with regular, non-commercial partners are consistently lower than the rates in commercial relationships [30, 31]. In this situation, partners of FEWs may potentially become a bridging population for HIV/STI transmission [12, 13]. Inconsistent condom use with sweethearts among FEWs could increase the HIV prevalence among the general population since their sweethearts may also have partners in the general population with whom they may not use condoms. Research shows that some Cambodian FEWs did not use condoms with clients for extra pay or by coercion [4]. Our findings of the factors associated with inconsistent condom use among FEWs fill in the gaps in the literature on sexual behaviors among FEWs in Cambodia, a high-risk population with complex relationships and sexual behaviors.

The successful decline in HIV prevalence in the Cambodian general population from 2.0% in 1998 to 0.6% in 2013 is widely attributed to the 100% Condom Use Program (CUP). The 100% CUP targets brothels as primary risk environments for HIV through multi-sector engagement and mobilization of local authorities, health workers, brothel owners, sex workers and community health workers to promote universal condom use and routine HIV and STI testing. The criminalization of sex work and brothels in 2008 has reversed some of the gains of the successful 100% CUP that was scaled up throughout Southeast Asia in the 1990s; new strategies are therefore urgently needed to address a persistent epidemic in sub-populations of women engaging in transactional sex [1]. The illegalization of sex work has made the 100% CUP approach infeasible because it entails explicit recognition of sex work by venues (brothels, massage parlors, karaoke bars, etc.), their managers and workers. As a result, sex work in Cambodia has transitioned to indirect transactional sex relationships known as entertainment work.

The finding that FEWs who had not received any form of HIV and SRH education were significantly more likely to report inconsistent condom use may reflect the effectiveness of education campaigns performed by outreach workers in community-based HIV/SRH integration programs in Cambodia such as the SAHACOM project [32]. Several satisfactory changes have been reported in the impact evaluation study of the SAHACOM; however, challenges remained in improving and sustaining the rates of consistent condom use, particularly in regular and non-commercial relationships [32]. Tailored education programs are required to respond to the needs of FEWs and their sweethearts.

In the SAHACOM project, where FEWs in this study were recruited, outreach workers led much of the project activities. Project activities included: (1) outreach sessions with FEWs at entertainment venues, which were led by trained peer outreach workers who used behavior change communication techniques to promote healthy sexual behaviors including condom use, HIV/STI testing, contraceptive use and other health services; (2) outreach workers offering workplace-based counseling and finger-prick HIV and STI testing to FEWs; case management including referrals for treatment at health facilities and (3) access to vocational centers for those seeking to pursue new professions.

We found that inconsistent condom users were more likely to report having been tested for HIV in the past six months. This finding may be supported by a number of health behavioral theories, including Protection Motivation Theory [33] and Health Belief Model [34], which view risk perception as an important determinant of healthcare seeking behaviors. FEWs who are involved in unprotected sex may choose to undergo HIV testing because of their perception of the risk they are involved, and they get HIV testing to confirm or rule out the possibility of the transmission. However, as shown in Table 1, HIV risk perception was not significantly associated with condom use with sweethearts among FEWs in this study, although the proportion of FEWs who perceived that their HIV risk was higher than that of the general population was higher among inconsistent condom users (20.3 vs. 29.3%). Moreover, our separate analysis of the same study sample did not find a significant association between HIV risk perception and HIV testing [35]. A study in China found that FSWs who had used HIV or STI services were more likely to use condoms consistently during commercial sex [18].

In this study, FEWs who used contraceptive methods other than condoms such as pills, injection, intrauterine devices, implant or natural methods were more likely to be involved in inconsistent condom use with sweethearts than those who used condoms as the main contraceptive method. The use of non-barrier contraception was also associated with inconsistent condom use with non-commercial partners among FSWs in Bolivia [17] and Swaziland [36]. Such practices may put FEWs at great risk for HIV and STI acquisition and transmission. This finding also underscores a challenging dilemma in HIV prevention where condom use is the most effective prevention method for HIV and STIs, yet women who opt to use highly effective contraceptive methods are less likely to use condoms. It also highlights the importance of programmatic promotion of dual protection method, using condoms in conjunction with other modern contraceptive methods that may increase protection against both HIV and unwanted pregnancies [36, 37]. However, consistent condom use remains the most feasible and effective dual protection strategy [17, 36].

We also found that the average amount of alcohol use per day in the past three months was high among FEWs, and the high alcohol consumption was significantly correlated with inconsistent condom use with sweethearts. This finding is in line with a Chinese study that highlighted heavy alcohol drinking among FSWs and its association with inconsistent condom use with both commercial and non-commercial partners [15]. Similarly, a Ugandan study unveiled that high alcohol use among FSWs was linked with unprotected sex with clients [16]. Therefore, alcohol use mitigation should be integrated into HIV prevention programs with FEWs. Further, given that alcohol drinking for many FEWs is job-related, alcohol consumption in entertainment establishments should be reckoned as an “occupational hazard” that warrants regular screening and intervention.

This study has some limitations. First, the self-reported measures may limit our findings through inherent biases, including both underreporting and over-reporting. Given the Cambodian cultural norms, it is likely that risky sexual behaviors and substance use among FEWs in this study were underreported [38]. Second, we included only FEWs from two provinces where the SAHACOM, a comprehensive community-based HIV/SRH integrated project, has been implemented for FEWs since 2009. In such condition, the levels of HIV risk and behaviors among FEWs in this study may not represent the situation of the general FEW population in other areas of Cambodia. The final limitation concerns the cross-sectional design of the study that did not allow us to draw causal relationships between the variables.

## Conclusions

Our findings highlight the low rate of consistent condom use in romantic relationships among FEWs. This situation puts FEWs at great risk for HIV and STI acquisition and transmission. In Cambodia, extensive progress has been made in the implementation of structural community-based HIV interventions with service packages, specifically designed for key populations, including FEWs. Further efforts are needed in order to increase condom use with sweethearts among these vulnerable women by addressing the key factors, including improving access to HIV and SRH education. The detrimental effects of multiple, concurrent partnerships and inconsistent condom use with sweethearts should be emphasized in education sessions and materials, particularly for FEWs who use non-barrier contraceptive methods.

## Abbreviations

AIDS: Acquired immunodeficiency syndrome
AOR: Adjusted odds ratio
ART: Antiretroviral therapy
C/PITC: Community/peer-initiated testing and counseling
CI: Confidence interval
CUP: Condom use program
FEWs: Female entertainment workers
FSWs: Female sex workers
GHQ: General Health Questionnaire
HIV: Human immunodeficiency virus
OR: Odds ratio
SAHACOM: Sustainable Action against HIV and AIDS in Communities
SD: Standard deviation
SRH: Sexual and reproductive health
STIs: Sexually transmitted infections

## References

[1] International Labor Organization (ILO). Cambodia - addressing HIV vulnerabilities of indirect sex workers during the financial crisis: Situation analysis, strategies and entry points for HIV/AIDS workplace education. Bangkok: ILO; 2011.

[2] Ministry of Justice (2011). Law on suppression of human trafficking and sexual exploitation 2008.

[3] Yi S, Tuot S, Yung K, Kim S, Chhea C, Saphonn V (2014). Factors associated with risky sexual behavior among unmarried most-at-risk young people in Cambodia. Am J Public Health Res.

[4] Ministry of Education, Youth and Sport (MoEYS) (2012). Examining Life Experiences and HIV Risks of Young Entertainment Workers in Four Cambodian Cities.

[5] Sopheab H, Tuot S, Chhea C, Gorbach P (2015). Characteristics, risk behaviors and factors associated with abortion among female entertainment workers in Cambodia. Reprod Health.

[6] Chhea C, Saphonn V (2011). Report on Estimation and Projections on HIV/AIDS in Cambodia 2010–2015.

[7] National Center for HIV/AIDS, Dermatology and STD (NCHADS) (2013). Standard Operating Procedure (SOP) for Boosted Continuum of Prevention to Care and Treatment for Most at Risk Populations in Cambodia.

[8] FHI 360 Cambodia (2011). Program Review: Providing HIV/AIDS Prevention and Care for Entertainment Workers (Reporting Period: October 2008–June 2010).

[9] Vun MC, Fujita M, Rathavy T, Eang MT, Sopheap S, Sovannarith S (2014). Achieving universal access and moving towards elimination of new HIV infections in Cambodia. J Int AIDS Soc.

[10] Couture MC, Sansothy N, Sapphon V, Phal S, Sichan K, Stein E (2011). Young women engaged in sex work in Phnom Penh, Cambodia, have high incidence of HIV and sexually transmitted infections, and amphetamine-type stimulant use: new challenges to HIV prevention and risk. Sex Transm Dis.

[11] Heng S, Tuot S (2013). Mid-term review of the sustainable action against HIV and AIDS in communities (SAHACOM).

[12] Yi S, Tuot S, Chhoun P, Brody C, Tith K, Oum S (2015). The impact of a community-based HIV and sexual reproductive health program on sexual and healthcare-seeking behaviors of female entertainment workers in Cambodia. BMC Infect Dis.

[13] Yi S, Tuot S, Chhoun P, Dyla PK, Tith K, Brody C (2015). Factors associated with induced abortion among female entertainment workers: A cross-sectional study in Cambodia. BMJ Open.

[14] Zhao J, Song F, Ren S, Wang Y, Wang L, Liu W (2012). Predictors of condom use behaviors based on the Health Belief Model (HBM) among female sex workers: a cross-sectional study in Hubei Province. China PLoS One.

[15] Chen Y, Li X, Zhang C, Hong Y, Zhou Y, Liu W (2013). Alcohol use and sexual risks: use of the Alcohol Use Disorders Identification Test (AUDIT) among female sex workers in China. Health Care Women Int.

[16] Bukenya J, Vandepitte J, Kwikiriza M, Weiss HA, Hayes R, Grosskurth H (2013). Condom use among female sex workers in Uganda. AIDS Care.

[17] Yam EA, Tinajeros F, Revollo R, Richmond K, Kerrigan DL, Garcia SG (2013). Contraception and condom use among Bolivian female sex workers: relationship-specific associations between disease prevention and family planning behaviors. Health Care Women Int.

[18] Gu J, Bai Y, Lau JT, Hao Y, Cheng Y, Zhou R (2014). Social environmental factors and condom use among female injection drug users who are sex workers in China. AIDS Behav.

[19] Aho J, Koushik A, Rashed S (2013). Reasons for inconsistent condom use among female sex workers: need for integrated reproductive and prevention services. World Health Popul.

[20] Deering KN, Bhattacharjee P, Bradley J, Moses SS, Shannon K, Shaw SY (2011). Condom use within non-commercial partnerships of female sex workers in southern India. BMC Public Health.

[21] National Institute of Public Health, National Institute of Statistics and ORC Macro (2010). Cambodia Demographic and Health Survey 2010.

[22] Yi S, Poudel KC, Yasuoka J, Ichikawa M, Tan V, Jimba M (2009). Influencing factors for seeking HIV voluntary counseling and testing among tuberculosis patients in Cambodia. AIDS Care.

[23] Yi S, Poudel KC, Yasuoka J, Palmer PH, Yi S, Jimba M (2010). Role of risk and protective factors in risky sexual behavior among high school students in Cambodia. BMC Public Health.

[24] Yi S, Poudel KC, Yasuoka J, Palmer PH, Yi S, Jimba M (2011). Risk vs. protective factors for substance use among adolescents in Cambodia. J Subst Use.

[25] Goldberg DP (1972). The detection of psychiatric illness by questionnaire: A technique for identification and assessment of non-psychotic psychiatric illness.

[26] Goldberg DP, Williams P (1988). A user’s guide to the General Health Questionnaire.

[27] Goldberg DP, Oldehinkel T, Ormel J (1998). Why GHQ threshold varies from one place to another. Psychol Med.

[28] Carey MP, Schroder KE (2002). Development and psychometric evaluation of the brief HIV Knowledge Questionnaire. AIDS Educ Prev.

[29] Family Health International (FHI). Behavioral Surveillance Surveys (BSS), Guidelines for repeated behavioral surveys in populations at risk of HIV: Chapter 5: Weighting in multi-stage sampling. Washington: FHI; 2000.

[30] Chow EP, Muessig KE, Yuan L, Wang Y, Zhang X, Zhao R (2015). Risk behaviours among female sex workers in China: a systematic review and data synthesis. PLoS One.

[31] Luchters S, Richter ML, Bosire W, Nelson G, Kingola N, Zhang XD (2013). The contribution of emotional partners to sexual risk taking and violence among female sex workers in Mombasa, Kenya: a cohort study. PLoS One.

[32] Yi S, Chhoun P, Brant S, Kita K, Tuot S (2014). The Sustainable Action against HIV and AIDS in Communities (SAHACOM): End-of-project evaluation.

[33] Rogers RW, Prentice-Dunn S, Gochman DS (1997). Handbook of health behavior research: Personal and social determinants.

[34] Rosenstock IM, Stretcher VJ, Becker MH (1994). The health belief model and HIV risk behavior change. In: Preventing AIDS: theories and methods of behavioral interventions.

[35] Brody C, Tuot S, Chhoun P, Pal K, Chhim K, Yi S. Recent HIV Testing and Associated Risk Factors among Female Entertainment Workers in Cambodia. Int J STD & AIDS. (In press).

[36] Yam EA, Mnisi Z, Mabuza X, Kennedy C, Kerrigan D, Tsui A (2013). Use of dual protection among female sex workers in Swaziland. Int Perspect Sex Reprod Health.

[37] Cates W, Steiner MJ (2002). Dual protection against unintended pregnancy and sexually transmitted infections: what is the best contraceptive approach?. Sex Transm Dis.

[38] UNFPA Asia Pacific Regional Office (2011). Socio-Cultural Influences on the reproductive health of migrant women: A review of literature in Cambodia, Lao PDR, Thailand and Viet Nam.

